# A Rare Case of Pulmonary Arterial Hypertension in a Patient With Breast Cancer Treated With Trastuzumab Emtansine

**DOI:** 10.7759/cureus.109066

**Published:** 2026-05-17

**Authors:** Abbie Maggs, Zeyad Elmarzouky, Chrysovalantou Nikolaidou

**Affiliations:** 1 Cardiology, Gloucestershire Royal Hospital, Gloucester, GBR

**Keywords:** cardio-oncology, drug-induced pulmonary hypertension, her2-targeted therapy, pulmonary arterial hypertension, trastuzumab emtansine (t-dm1)

## Abstract

Breast cancer is the most common malignancy in women worldwide. A large proportion of breast cancer cases demonstrate human epidermal growth factor receptor 2 (HER2) overexpression. Trastuzumab emtansine (T-DM1) is an established therapy for HER2-positive metastatic breast cancer, though rare but serious adverse effects may occur. We report a 58-year-old woman with HER2-positive metastatic breast cancer who developed progressive exertional dyspnoea while receiving T-DM1. Echocardiography demonstrated right ventricular dilation and dysfunction with elevated pulmonary pressures. Right heart catheterisation confirmed pulmonary arterial hypertension (PAH) with increased pulmonary vascular resistance. Comprehensive evaluation, including computed tomography, pulmonary angiography, and ventilation-perfusion scanning, excluded thromboembolic disease. There was no significant parenchymal lung disease, and left ventricular function was preserved.

In the absence of alternative causes and given the temporal relationship with T-DM1 exposure, drug-induced PAH was considered likely. The patient was treated with sildenafil and ambrisentan, resulting in symptomatic and haemodynamic improvement. Concurrent hepatotoxicity also resolved following discontinuation of T-DM1, further supporting a causal association. PAH is a rare complication of HER2-targeted therapies, with limited cases reported. This case highlights the importance of recognising PAH as a potential adverse effect of T-DM1. Clinicians should maintain a high index of suspicion in patients presenting with unexplained dyspnoea or right ventricular dysfunction to enable early diagnosis and appropriate management.

## Introduction

Breast cancer is the most common malignancy in women worldwide and represents the second most common cancer overall, accounting for approximately 11.6% of all cancer diagnoses [1,2]. In 2022, around 2.3 million women were diagnosed with the disease, with a total of 666,000 deaths globally [2]. Approximately 15-20% of patients with breast cancer exhibit amplification or overexpression of human epidermal growth factor receptor 2 (HER2). Under physiological conditions, HER2 signalling promotes normal cellular growth and division. However, when overexpressed, the pathway is exploited and promotes rapid cell proliferation and tumour formation and progression [3]. The overexpressed HER2 receptor has become an important therapeutic target. Anti-HER2 therapies have led to improved survival, even in the metastatic setting. First-line treatment for HER2-positive metastatic breast cancer typically includes dual anti-HER2 therapy with trastuzumab and pertuzumab in combination with a taxane [4]. If disease progresses despite this optimal therapy, second-line treatment includes trastuzumab emtansine (T-DM1) or trastuzumab deruxtecan [5]. T-DM1 is an antibody drug conjugate consisting of a monoclonal antibody (trastuzumab) covalently linked to the cytotoxic agent DM1. T-DM1 binds to HER2-overexpressing tumour cells, triggering receptor-mediated internalisation and lysosomal degradation, which leads to mitotic arrest and cell death [6].

Common side effects of T-DM1 are fatigue, nausea, and vomiting. More severe side effects include thrombocytopenia, neutropenia, and leukopenia. Pneumonitis of mild or moderate severity was identified in 1.9% of patients, and a reduction of left ventricular systolic function was reported in 0.4% of patients. No cardiac failure or incidence of pulmonary arterial hypertension (PAH) was reported [7]. After an extensive search of the literature, only two case reports were identified linking T-DM1 to PAH [8,9]. We present a case of a patient with metastatic breast cancer who developed PAH on treatment with T-DM1.

## Case presentation

A 58-year-old woman was referred to our cardio-oncology clinic due to changes in the right ventricular size and function detected on follow-up echocardiographic imaging. She was diagnosed with HER-2-positive breast cancer with bone metastases in 2008. She was initially treated with docetaxel and fluorouracil, epirubicin hydrochloride, and cyclophosphamide (FEC) chemotherapy. Following chemotherapy, she underwent a left total mastectomy with lymph node clearance and received adjuvant radiotherapy. She then started treatment with trastuzumab. This was switched to T-DM1 in 2017 due to the progression of metastatic bone disease. She was also on treatment with tamoxifen, denosumab, and antihypertensive medications. She was an ex-smoker (35-year pack-year smoking history) but had no history of lung disease. She denied recreational drug use.

**Figure 1:**
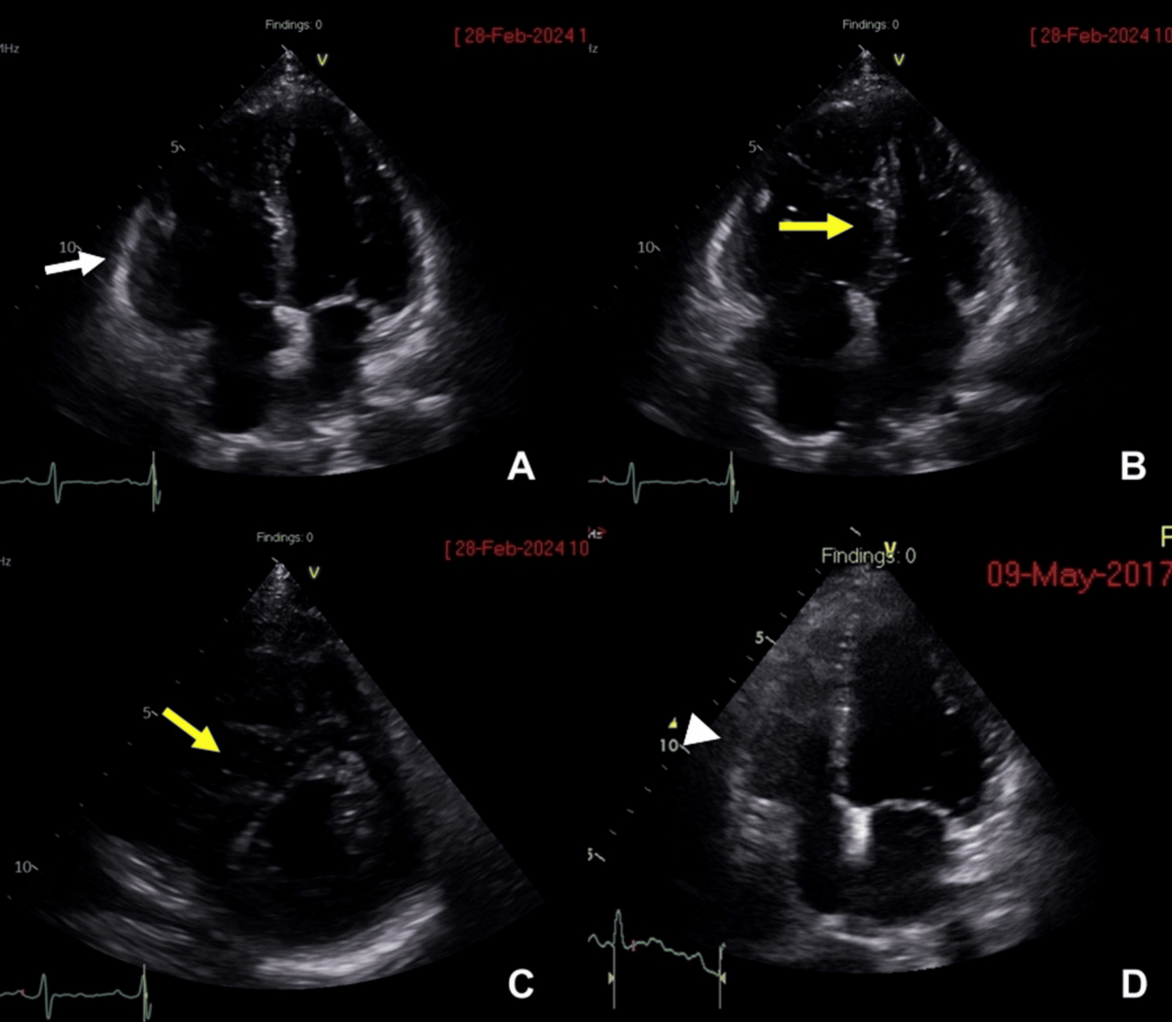
Transthoracic echocardiogram showing the dilated right ventricle An apical four-chamber right ventricle (RV) focused view (white arrow, panel A), with systolic flattening of the septum on the apical four-chamber view and parasternal mid-ventricular short-axis view (yellow arrow, panels B and C), in keeping with RV systolic pressure overload. Panel D shows normal RV dimensions with an RV basal diameter of 29 mm on apical four-chamber view (white arrowhead) on echocardiogram performed in 2017, prior to commencing treatment with trastuzumab emtansine

**Figure 2:**
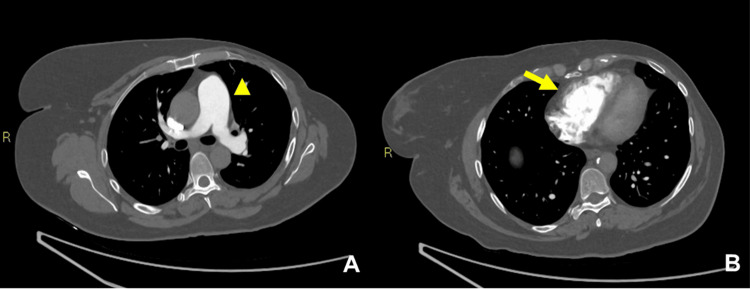
CT pulmonary angiogram to exclude pulmonary embolism CT pulmonary angiogram showing the dilated main pulmonary artery (yellow arrowhead, Panel A) with no pulmonary emboli in the proximal pulmonary artery branches, and the dilated right ventricle (yellow arrow, Panel B). Left mastectomy with absence of left breast tissue

The patient reported progressively worsening exertional dyspnoea over the preceding six months and denied other cardiac symptoms. Clinical examination was unremarkable. Electrocardiography demonstrated a sinus rhythm with T-wave inversion in the anterior leads. The patient had raised hepatic enzymes and was under investigation for hepatic fibrosis. There was no evidence of cirrhosis or porto-pulmonary hypertension. Viral hepatitis screening was negative. The N-terminal pro-B-type natriuretic peptide (NT-proBNP) was normal. Her recent echocardiogram showed a dilated right ventricle (RV) (basal diameter, 46 mm; mid-RV diameter, 42 mm; length, 71 mm) (Figure 1A) with mildly impaired systolic function (tricuspid annular plane systolic excursion (TAPSE), 16 mm; FAC, 33%). The estimated RV systolic pressure was elevated (RVSP, 50 mmHg), with systolic septal flattening and a D-shaped left ventricle consistent with RV pressure overload (Figures 1B-1C). LV size and systolic function were normal. Only mild tricuspid regurgitation was present. Baseline RVSPs were not measured, as RV size and systolic function were normal on previous echocardiograms. Basal RV diameter was 29 mm and TAPSE 17 mm on the echocardiogram prior to commencing treatment with T-DM1 (Figure 1D). Pulmonary function testing demonstrated reduced lung volumes: forced expiratory volume at one second (FEV1), 1.73 L (68% predicted); forced vital capacity (FVC), 2.31 L (71% predicted); transfer factor of the lung for carbon monoxide (TLCO), 70% predicted; and carbon monoxide transfer coefficient (KCO), 96% predicted. A CT pulmonary angiogram showed minor radiotherapy changes, a few isolated lung cysts, and no evidence of thromboembolic disease (Figure 2). The RV was apex forming, and the main pulmonary artery (PA) was enlarged (40 mm in diameter). Serological testing for connective tissue disease was unremarkable, and there were no clinical features suggestive of connective tissue disease. Treatment with bumetanide was commenced, and the patient was referred to a specialised pulmonary hypertension (PH) clinic in a tertiary centre. A subsequent ventilation-perfusion (VQ) scan completely excluded thromboembolic disease. Right heart catheterisation confirmed PAH with the following haemodynamic parameters: RVSP, 70 mmHg; pulmonary artery pressure, 72/28 mmHg (mean, 44 mmHg); mean right atrial pressure, 7 mmHg; pulmonary capillary wedge pressure (PCWP), 10 mmHg (end-expiratory); transpulmonary gradient, 34 mmHg; and pulmonary vascular resistance (PVR), 8.29 Wood units. Vasodilator challenge was negative. She was started on combination treatment with a PDE5 inhibitor, sildenafil, and ambrisentan. The patient’s symptoms and six-minute walk distance improved after four months of treatment. A repeat right heart catheterisation nine months on treatment demonstrated improvement in pressures: RV systolic pressure, 55 mmHg; PA pressure, 54/18; mean 35 mmHg; mean right atrial pressure, 7 mmHg; PCWP end-expiratory, 18 mmHg; transpulmonary gradient, 17 mmHg; and PVR, 2.7 Wood units.

## Discussion

In the absence of alternative causes of PH or significant lung disease associated with the malignancy, two potential aetiologies of the PH were considered. Either the patient incidentally developed idiopathic PAH, or this was caused by the treatment with T-DM1. Although PAH in the patient can be coincidental, the temporal relationship between the exposure to T-DM1 and the clinical manifestation of symptoms supports a possible causal association.

Drug-induced PAH is classified as Group 1 PH and has been reported with several medications, including targeted cancer therapies [10]. PAH associated with HER2-targeted therapy is rare. We found only two other cases of PAH in patients treated with T-DM1. The first case involved a 43-year-old woman who developed PAH and haemorrhagic telangiectasia after six cycles of treatment [8]. The second case was a 36-year-old female patient who developed shortness of breath after 19 months of treatment with T-DM1. The authors of this case report queried the FDA Adverse Event Reporting System (FAERS) Pharmacovigilance Database and found 22 reported cases of PAH associated with the use of HER2-targeting therapies. They subsequently conducted a disproportionality analysis by calculating the reported odds ratio of PAH with HER2-targeted treatments and found six cases associated with T-DM1 as the potential cause. The signal of trastuzumab and pertuzumab for PAH was weak based on the reported odds ratio [9]. Another pharmacovigilance database disproportionality analysis, which used data from VigiBase, the largest pharmacovigilance worldwide database, identified four cases of PAH in patients on T-DM1 treatment with a mean time to onset of 201 days. Additionally, eight cases of PAH associated with trastuzumab use were identified, with a mean time to onset of 562 days [11]. A French study from 2024 extracted data about the incidence of PAH cases from the French PH registry and identified eight cases treated with T-DM1 [12]. The mechanism of PAH caused by T-DM1 is unclear. Microtubule disruption, apoptosis of potentially HER2-expressing endothelial cells, and endothelial injury with T-DM1 have been proposed as potential mechanisms. It has been hypothesised that PAH is mediated via the ACVRL1 mutation pathway [9].

## Conclusions

This case highlights a rare occurrence of PAH likely secondary to treatment with T-DM1. Clinicians managing patients receiving HER2-targeted therapies should be aware of the potential risk of PAH, particularly in patients presenting with unexplained dyspnoea or echocardiographic evidence of right ventricular dysfunction. Early recognition of these patients and subsequent referral to specialist PH centres can facilitate timely diagnosis and management.
